# Evaluation of the In Vitro Biocompatibility of PEDOT:Nafion Coatings

**DOI:** 10.3390/nano11082022

**Published:** 2021-08-08

**Authors:** Sonia Guzzo, Stefano Carli, Barbara Pavan, Alice Lunghi, Mauro Murgia, Michele Bianchi

**Affiliations:** 1 Center for Translational Neurophysiology, Istituto Italiano di Tecnologia, 44121 Ferrara, Italy; sonia.guzzo@iit.it (S.G.); crlsfn@unife.it (S.C.); pvnbbr@unife.it (B.P.); alice.lunghi@iit.it (A.L.); mauro.murgia@iit.it (M.M.); 2 Department of Neuroscience and Rehabilitation, Section of Physiology, University of Ferrara, 44121 Ferrara, Italy; 3 Department of Chemical, Pharmaceutical and Agricultural Sciences, University of Ferrara, 44121 Ferrara, Italy; 4 Institute of Nanostructured Materials (ISMN), National Research Council (CNR), 40129 Bologna, Italy

**Keywords:** conducting polymers, organic bioelectronics, surface coating, cell adhesion, cell proliferation, atomic force microscopy, scanning electron microscopy, in vitro cytotoxicity

## Abstract

Poly(3,4-ethylenedioxythiophene)-Nafion (PEDOT:Nafion) is emerging as a promising alternative to PEDOT-polystyrene sulfonate (PEDOT:PSS) in organic bioelectronics. However, the biocompatibility of PEDOT:Nafion has not been investigated to date, limiting its deployment toward in vivo applications such as neural recording and stimulation. In the present study, the in vitro cytotoxicity of PEDOT:Nafion coatings, obtained by a water-based PEDOT:Nafion formulation, was evaluated using a primary cell culture of rat fibroblasts. The surface of PEDOT:Nafion coating was characterized by Atomic Force Microscopy (AFM) and water contact angle measurements. Fibroblasts adhesion and morphology was investigated by scanning electron microscopy (SEM) and AFM measurements. Cell proliferation was assessed by fluorescence microscopy, while cell viability was quantified by 3-(4,5-Dimethylthiazol-2-yl)-2,5-Diphenyltetrazolium Bromide (MTT), lactate dehydrogenase (LDH) and neutral red assays. The results showed that PEDOT:Nafion coatings obtained by the water dispersion were not cytotoxic, making the latter a reliable alternative to PEDOT:PSS dispersion, especially in terms of chronic in vivo applications.

## 1. Introduction

Poly(3,4-ethylenedioxythiophene):poly(styrenesulfonate) (PEDOT:PSS) is the most studied conducting polymer in the field of organic electronics. Its applications range from active layer in solar cells and organic light emitting diodes, mixed ionic–electronic conductor in organic electrochemical transistors and multielectrode arrays (MEAs), charge storage material in supercapacitors and antistatic layer in photo films and shielding coatings [1,2,3,4]. Its popularity is based on its high conductance and capacitance [5], stability, mixed ionic–electronic conduction [6] and possibility to be used as a disposable due to the presence of several commercial aqueous formulations [7]. For bioelectronic and neuroelectronic applications, where organic electronic devices operate as interfacial transducers between biological signals and electronic hardware, the biocompatibility of the conductive polymer is an important requirement, especially when chronic applications are envisioned [8]. PEDOT:PSS is undoubtedly a commodity, being available in several commercial formulations, and thus suitable for a number of microfabrication techniques such as inkjet printing, spin coating, 3D printing and casting, to cite a few [9,10]. However, there is usually an inverse correlation between PEDOT:PSS conductivity grade and biocompatibility, where the former property can be dramatically increased by secondary doping at the expense of its biocompatibility [7]. In addition, the adhesion of pristine PEDOT:PSS to common substrates such as glass or metals is usually rather low [11]. Therefore, silanes such as (3-glycidyloxypropyl)trimethoxysilane (GOPS), which partially improves a material’s conductivity and biocompatibility, are commonly added to ensure sufficient mechanical stability of the coating [12]. For the above reasons, alternative PEDOT formulations with high conductivity, adequate biocompatibility and that do not require secondary dopants or additives are highly desired in the field of organic bioelectronics.

We have recently proposed PEDOT doped with Nafion as a promising alternative to PEDOT:PSS in organic bioelectronics [13]. It is noteworthy that electrodeposited PEDOT/Nafion, in view of its higher capacitance and capability to act as an ion exchanger compared to electrodeposited PEDOT/PSS, exhibits a minimum polarization during electrical stimulation of less than a millisecond, resulting in a charge injection limit that is ca. 80% larger than the most widely used conductive polymer, making it a good candidate for both neural recording and stimulation [13]. Furthermore, we have recently reported a water-based (i.e., chemically synthesized) formulation of PEDOT:Nafion characterized by rapid ion transport and stability to delamination, even in the absence of adhesion promoters [14]. Due to the ability to be easily processed by wet techniques such as spin coating or drop casting, this formulation has been successfully integrated into the fabrication of state-of-the-art electrochemical organic transistors (EGOTs) and neuromorphic devices [14]. However, the biocompatibility of the water-based formulation of PEDOT:Nafion has not been investigated to date, effectively limiting its current use in long-term applications such as chronic neural recording and stimulation sessions. Therefore, in this study, we investigated the in vitro cytotoxicity of PEDOT:Nafion coatings using a primary cell culture of rat fibroblasts as a necessary go/no-go decision step toward future in vivo testing.

## 2. Materials and Methods

### 2.1. Synthesis of PEDOT:Nafion and Coating Fabrication

PEDOT:Nafion water dispersion was synthesized according to previously reported protocol [14]. The obtained water dispersion of PEDOT:Nafion (1 mL) was diluted with water (5 mL), and the mixture was sonicated at room temperature for 15 min. This treatment is necessary to reduce the aggregates in order to give an optimal deposition of PEDOT:Nafion. Subsequently, coatings were fabricated by drop casting 30 µL of the PEDOT:Nafion dispersion with a pipette on borosilicate glass slides (1 × 1 cm^2^, thickness ~ 1 mm, average root mean square (RMS) roughness: (0.21 ± 0.02) nm, Thermo Fisher Scientific, Milan, Italy) previously cleaned with an isopropyl alcohol, ethanol and water (1:1:1 vol%) solution and dried with N_2_. Subsequently, coatings were annealed at 120 °C for 40 min.

### 2.2. Surface Characterization of PEDOT:Nafion Coatings

Surface topography of PEDOT:Nafion coatings was investigated by atomic force microscopy (AFM). Images were acquired using a Park XE7 AFM System (Park Systems, Suwon, Korea) operating in tapping mode in air at room temperature. Premounted silicon cantilevers were used (OMCL-AC160TS, tip curvature radius ~ 7 nm, k ~ 26 N/m and Al backside coating, Olympus, Tokyo, Japan). The RMS was extracted from several topography images acquired at different scan size, i.e., from 1 × 1 µm^2^ to 10 × 10 µm^2^, using the XEI Software (XEI, version 4.3; Park Systems, Suwon, Korea, 2016). All images were previously flattened using a 2nd order regression in *x* direction and a 1st order regression in Y direction. All values are reported as mean ± standard deviation.

Wettability of PEDOT:Nafion coatings was investigated by a home-built water contact angle measurement unit. The value of the contact angle was obtained by averaging several measurements of the left and right contact angle of the water drop taken on different areas of the samples by using the ImageJ free software (https://imagej.nih.gov/).

### 2.3. Isolation and Culture of Primary Rat Fibroblasts

Fibroblasts were isolated from tail of Long–Evans rat (Charles River Laboratories, Lecco, Italy) during routine surgery and the seeded for culture. Experiments were performed in compliance with the guidelines established by the European Communities Council (Directive 2010/63/EU, Italian Legislative Decree n. 26, 4 March 2014), and the protocol was approved by the Ethics Committee for animal research of the University of Ferrara and by the Italian Ministry of Health (protocol n. 989/2020-PR, date 15 October 2020). Briefly, specimens (~1 cm) from scarification of the tail tip were obtained after skin sterilization with 70% *v*/*v* ethanol/water. Specimens were further washed in phosphate buffer saline (PBS, pH 7.4) and minced by scissors. Homogenate tissue was transferred into a sterile conical bottom tube and digested by an enzymatic mixture of 5 mg/mL Dispase and 200 UI/mL Collagenase in an orbital shaker at 200 rpm for 90 min at 37 °C. Digested homogenate was ground through a 70 µm cell strainer using a 10 mL syringe plunger. Cells were washed out of the strainer by means of Advance Dulbecco’s modified Eagle’s medium (DMEM), containing 10% *v/v* Fetal Bovine Serum (FBS), 200 µg/mL L-glutamine, 200 µg/mL penicillin/streptomycin and 250 µg/mL Amphotericin B, and cultured in petri dishes of a surface area of 100 × 20 mm^2^ in an atmosphere of 5% CO₂ and at 37 °C. Growth medium was changed every 3 days, and at confluence, the cells were trypsinized and harvested for biocompatibility assay. All the reagents for cell culture were purchased from Thermo Fisher Scientific (Milan, Italy).

### 2.4. Cells Culture on PEDOT:Nafion Coatings

Before cellular studies, PEDOT:Nafion coatings were sterilized in 70% *v/v* ethanol/water solution and dried under laminar flow hood. Then, cells were seeded at a density of 1 × 10^4^ cells per well on the PEDOT:Nafion coatings put in a 24-well culture plate (Corning^®^ Costar^®^ TC-Treated Multiple Well Plates, Merck Life Science S.r.l., Milan, Italy) and grown in 0.5 mL of complete DMEM for the selected experimental times (see below). Bare plastic dishes (Corning^®^ Costar^®^ TC-Treated Multiple Well Plates, Merck Life Science S.r.l., Milan, Italy) were used as control, unless otherwise specified. We preferred not to include here the PEDOT:PSS dispersion as control group, as the main aim was to collect preliminary evidence on the biocompatibility of conductive coatings obtained from a novel PEDOT:Nafion dispersion rather than compare the latter with a PEDOT:SS dispersion. In addition, different water-based PEDOT:PSS formulations are available, containing additives that can dramatically (positively or negatively) alter the final outcome, hindering a true comparison between different materials [7].

### 2.5. Evaluation of Cell Adhesion by Scanning Electron Microscopy (SEM) and AFM Investigations

Scanning electron microscopy (SEM) was used to qualitatively evaluate morphology of fibroblasts seeded on either PEDOT:Nafion coatings. Before analysis, samples were fixed in 2.5% glutaraldehyde in 0.1 M phosphate buffer (pH 7.4, Merck Life Science S.r.l., Milan, Italy), postfixed in 2% osmium tetroxide in the same buffer, dehydrated in an ascending series of alcohols and dried with hexamethyldisilazane. Samples were then mounted on a metal holder and gold coated (~15 nm) with a Q150RS magnetron sputter (QuorumTech, London, UK). A SEM ZEISS EVO40 XVP (Carl Zeiss NTS GmbH, Oberkochen, Germany) was used, operating at 20 kV acceleration voltage. Circular glass microcoverslips (uncoated glass slides, Waldemar Knittel Glasbearbeitungs GmbH, Braunschweig, Germany) were used as control. Cells attached to the respective substrate were rinsed twice with PBS and fixed with 3% glutaraldehyde in PBS for 45 min. After washing with PBS, dehydration was performed by slow water replacement using series of ethanol/water solution (30%, 50%, 70%, 90%) for 15 min with final dehydration in absolute ethanol for 30 min. AFM was used to gain additional insights about cells adhesion on PEDOT:Nafion coatings and glass substrate (see Section 3.2). However, for this purpose, no imaging flattening was carried out to preserve correct imaging of cell morphology.

### 2.6. Cell Viability and Proliferation

To evaluate cell proliferation, 2 × 10^4^ cells were seeded on PEDOT:Nafion coating and on control group. Fibroblasts viability was analyzed after 1, 3, 5 and 7 days in culture by staining with fluorescein diacetate (15 µg/mL FDA, green), propidium iodide (5 µg/mL PI, red) and Hoechst 33342 (10 µg/mL, blue) dissolved in 1x Ringer–Locke solution and incubation of 10 min at room temperature. Green and red fluorescent cells stain live and dead cells, respectively, and blue stains cell nuclei. After incubation, samples were washed twice with 1x Ringer–Locke solution, and their staining was acquired using an Olympus BX51 fluorescent microscope (MBF Bioscience, Williston, VT, USA) equipped with X-Cite 120 fluorescence illumination system (EXFO, Quebec, QC, Canada) and a color digital camera (MBF Bioscience, Williston, VT, USA).

### 2.7. Mitochondrial Function Studies

3-(4,5-Dimethyl-Thiazol-2-yl)-2,5-Diphenyltetrazolium Bromide (MTT) assay (Thermo Fisher Scientific, Milan, Italy) was used to evaluate the reduction-oxidation status of living cells and mitochondrial activity, reflecting cell survival due to the formation of formazan. The absorbance at 560 nm is a measure of the amount of red formazan dye produced by the reduction of yellow tetrazolium salt. The latter is converted by the tetrazolium ring cleavage by succinate dehydrogenase within the mitochondria [15]. Cells were seeded at a density of 2 × 10^4^ cells/well in 24-well plates over PEDOT:Nafion coatings and tested after 1 day and 7 days. After these times, 100 µL of MTT solution (5 mg/mL in PBS) were added to each sample and incubated for 4 h at 37 °C under a CO_2_ (5%) atmosphere. The medium was aspirated and 100 µL of dimethylsulfoxide (DMSO) were added to each well for 45 min at 37 °C upon a plate shaker. Each sample were transferred in a 96-well plate to read the absorbance at 560 nm using a multimode plate reader spectrophotometer (VICTOR X4, PerkinElmer, Waltham, MA, USA).

### 2.8. Lactate Dehydrogenase (LDH) Measurement

Cells were seeded at a density of 2 × 10^4^ cells/well in 24-well plates over PEDOT:Nafion coatings and assayed after 1 day and 7 days. LDH activity was measured CyQUANT™ LDH Cytotoxicity Assay Kit (Thermo Fisher Scientific, Milan, Italy) at 490 nm absorbance, according to the manufacturer’s protocol. The absorbance at 490 nm of the amount of red formazan dye is produced by the reduction of yellow tetrazolium salt. In this assay, the formation of formazan is mediated by NADH during the conversion of lactate to pyruvate [16]. Medium from each experimental condition was transferred into a 96-well plate in triplicate and incubated with the reaction mixture at room temperature for 30 min. The cytotoxicity of PEDOT:Nafion was calculated as: % Cytotoxicity = [(Compound-treated LDH activity − Spontaneous LDH activity)/(Maximum LDH activity − Spontaneous LDH activity)] × 100.

### 2.9. Neutral Red Uptake Assay

Cells were seeded at a density of 2 × 10^4^ cells/well in 24-well plates over PEDOT:Nafion coatings and assayed after 1 day and 7 days. Each sample was rinsed with PBS, and 100 µL of 100 µg/mL neutral red solution dissolved in culture medium containing 5% FBS were added to each well. After a 3 h of incubation at 37 °C, neutral red solution was removed, and dye extraction was performed by adding 100 µL of 1% *v/v* acetic acid in 50% *v/v* ethanol/water solution into each well. The plates were gently shaken for 10 min, and the absorbance was measured at 540 nm. The absorbance at 490 nm is a measure of the amount of red formazan dye produced by the reduction of yellow tetrazolium salt. In this assay, the formation of formazan is mediated by NADH during the conversion of lactate to pyruvate [17].

### 2.10. Data Analysis

Graphs and data were plotted and analyzed by software GraphPad (*GraphPad*, version Prism 6, GRAPHPAD 2365 Northside Dr., San Diego, CA, USA, 2102). Statistical analysis was performed by one-way analysis of variance (ANOVA) followed by Bonferroni’s post-test and unpaired t-test for statistical comparison between control and materials tested in several assays, with the significance level set at *p*-value < 0.05. Data were expressed as means ± standard deviations of experiments carried out in triplicate.

## 3. Results and Discussion

### 3.1. Surface Characterization of PEDOT:Nafion Coatings

**Figure 1:**
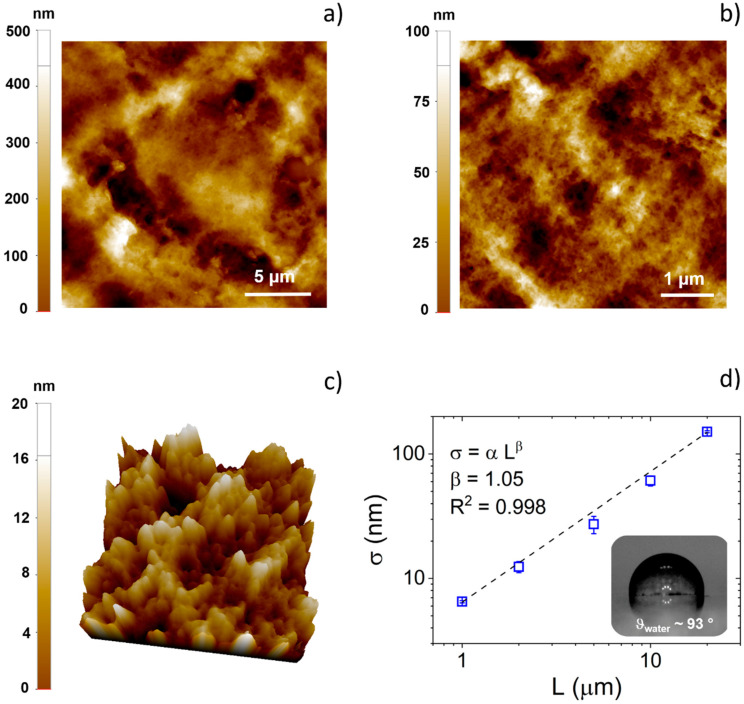
Surface characterization of PEDOT:Nafion coatings. Two-dimensional (**a**,**b**) and three-dimensional (**c**) topography images of PEDOT:Nafion coatings acquired at different scale lengths. The lateral scan size is 1 µm in (c). (**d**) Plot of RMS (σ) vs. lateral scan size (L). The dashed dot size represents the best fit, according to the power law (σ = α L^β^). Inset of (d): a representative image of a water droplet on the PEDOT:Nafion coating.

AFM images of the surface of the PEDOT:Nafion coating deposited by drop casting on glass substrate are shown in Figure 1. In Figure 1a,b, topography images of the conducting polymer coating acquired at relatively large scan size (20 × 20 and 5 × 5 µm^2^) pointed out a poorly ordered and highly rough surface, typical of polymeric coatings deposited by direct casting techniques [18,19]. However, as can be observed in Figure 1c, on a smaller length scale, PEDOT:Nafion coating showed a nanostructured surface, featuring nanograins of the average size of ca. 30–50 nm, comparable to that of drop casted films of the most used water-based formulation of PEDOT:PSS (i.e., Clevios PH1000) [14]. It should be noted that the presence of a nanostructured surface is generally beneficial for bioapplications, as submicrometer- and nanometer-sized features are well known to be highly effective in modulating actin cytoskeleton dynamics and cell-adhesion receptors, respectively [20,21,22,23].

Quantitative evolution of the RMS (σ) with the image scan size (L) is reported in Figure 1d. As previously reported for spun-coated PEDOT:PSS films [5], RMS data could be fitted with a power law fit σ = α L^β^, where α is the prefactor and β is the scaling exponent [24,25,26]. However, in this case, the absence of the horizontal upper cutoff within the investigated scan size range indicates that the casted PEDOT:Nafion coatings were highly rough, even at large length scales (i.e., >20 µm), the length scales that are of interest for single cell or cell population dynamics [20,27,28]. Finally, it can be noted that the roughness of PEDOT:Nafion coatings deposited by drop casting is much larger not only of that of PEDOT:PSS films realized by spin coating, as one may expect (only few nanometers at 15 × 15 µm^2^ of scan size) [5], but also of that of electrodeposited PEDOT:Nafion coatings (ca. 70 nm at 15 × 15 µm^2^ of scan size) [13].

As a direct consequence of both the high surface roughness and of the hydrophobic polytetrafluoroethylene backbone featuring regularly spaced perfluorovinyl ether side-chains of the Nafion polymer, the water contact angle of PEDOT:Nafion coatings resulted in being quite high (93 °, see inset of Figure 1d), typical of a moderately hydrophobic material.

### 3.2. Cell Adhesion and Proliferation on PEDOT:Nafion

**Figure 2:**
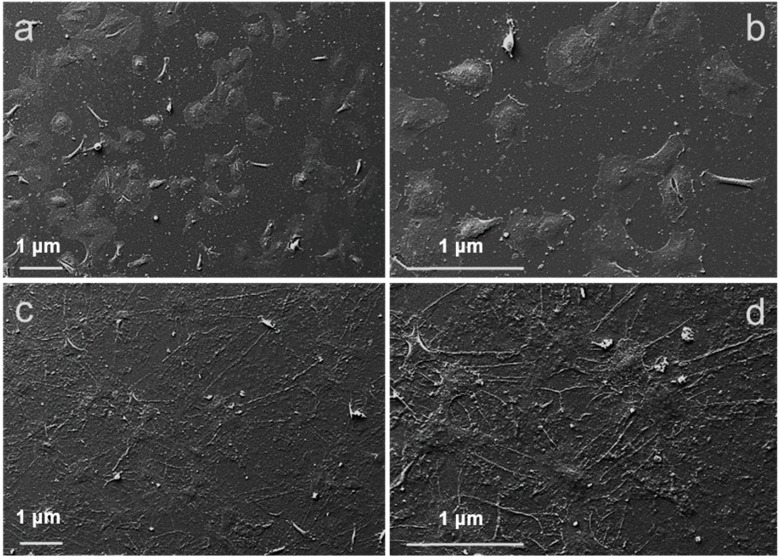
SEM investigation of primary fibroblasts adhesion on glass control and on PEDOT:Nafion. Morphology of cells seeded on control glass (**a**,**b**) and PEDOT:Nafion coatings (**c**,**d**) at 12 h after seeding at a density of 1 × 10^4^ cells/cm^2^. Panels (**b**,**d**) magnified images from (**a**,**c**), respectively.

Morphology of fibroblasts adhered on PEDOT:Nafion coating and on the glass control substrate at 12 h after seeding was initially evaluated by SEM analysis (Figure 2). Fibroblasts adhered on the control glass showed a rounded shape, few intracellular connections and a limited coverage of the substrate, indicating that, at 12 h from seeding, cells are still in an initial stage of adhesion (Figure 2a,b). By contrast, fibroblasts adhered on PEDOT:Nafion coating showed a flattened morphology, extended intracellular connections, full coverage of the substrate and well-developed filopodia, suggesting a more advanced stage of adhesion compared to cells on glass (Figure 2c,d).

**Figure 3:**
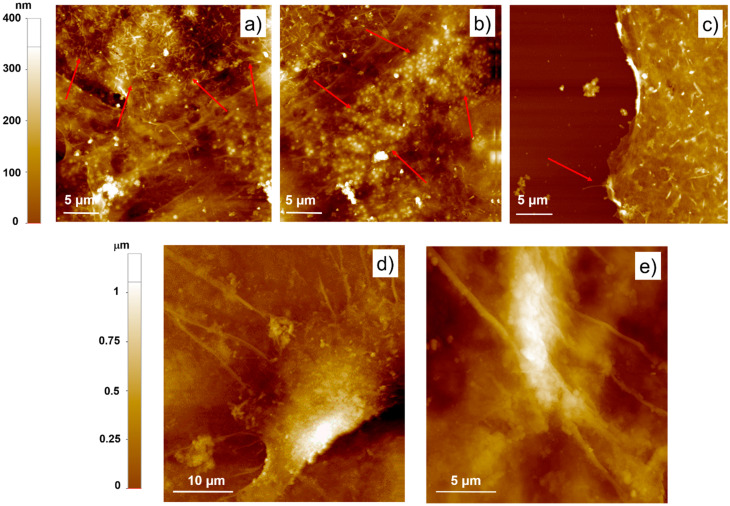
AFM characterization of fibroblasts adhered to glass substrate and PEDOT:Nafion. AFM topography images of primary rat fibroblasts after 12 h from seeding on control glass (**a**–**c**) and PEDOT:Nafion (**d**,**e**) substrates. In (**a**,**b**), the actin filaments of the cell cortex and the round membranous vesicles (indicated by red arrows) are shown, respectively. In (**c**), a detail of a typical cell lamellipodium characterized only by small and rare filopodia (marked by the arrow) is shown. In (**d**,**e**), two different well-adhered fibroblasts on PEDOT:Nafion coating and showing well-developed filopodia are shown.

Further insights regarding cell adhesion could be obtained by AFM analysis (Figure 3). Interestingly, topography images pointed out the presence of the cell cortex (Figure 3a), as previously reported by scanning force microscopy studies [29,30]. The cell cortex, also called actin cortex, is a thin and dense network of actomyosin under the plasma present in most mammalian cells [31]. The main role of the actin cortex is to guide cell shape changes required for cell adhesion, migration, division and tissue morphogenesis. Therefore, an abundant presence of this structure suggests that the cell is in an active growth phase and is still adapting its morphology to the substrate [31,32]. Furthermore, AFM images of fibroblasts seeded on glass also revealed the presence of areas accumulating numerous round-shaped membranous vesicles and an underlying mat of less dense actin filament than the actin cortex (Figure 3b). According to the literature, it is, therefore, possible to assume that these vesicles are zones of active production of the actin cortex [33]. Finally, as yet observed by SEM analysis, adherent fibroblasts on glass were characterized by the presence of bare lamellopodia, with only initial or even absent filopodia (Figure 3c). In contrast, no obvious cell cortex could be identified in the fibroblasts adhered on the PEDOT:Nafion coatings at 12 h form seeding (Figure 3d,e). This observation, together the presence of well-developed filopodia compared to cells adhered on glass, suggested that cells seeded on PEDOT:Nafion were in a more advanced state of adhesion than cells on glass substrate at the selected experimental time point.

It is widely reported that micro/nanoroughness and hydrophilicity/hydrophobicity can strongly influence cell adhesion and proliferation [22,34,35]. Indeed, coatings exhibiting a rough surface texture are expected to improve cell adhesion and spreading compared to smooth ones [21,36]. This can be attributed to the larger surface area offered by rough coatings, which promotes protein adhesion, the latter being the first event after the implantation of any material in the human body [34]. In this view, the combined large scale and nanoscale roughness showed by the PEDOT:Nafion coating strongly promoted the adhesion of the fibroblasts compared to the smooth glass substrate. Regarding surface wettability, moderate or highly hydrophilic surfaces are expected to promote cell adhesion and proliferation compared to highly hydrophobic surfaces [28,34,37]. Therefore, the rather large hydrophobicity of the PEDOT:Nafion (contact angle of ~93°), would be expected to discourage cell adhesion compared, for instance, to the moderately hydrophilic surface of the control (contact angle of ~39°, data not shown). However, this is not the case, as fibroblasts adhered more rapidly on PEDOT:Nafion coating than on the control. It can be therefore concluded that the much higher micro/nanoroughness of casted PEDOT:Nafion (RMS on a 5 × 5 μm scale ~ 20 nm) compared to glass (RMS on a 5 × 5 μm scale ~ 0.2 nm) was instrumental in promoting fibroblast adhesion on the conductive polymer.

**Figure 4:**
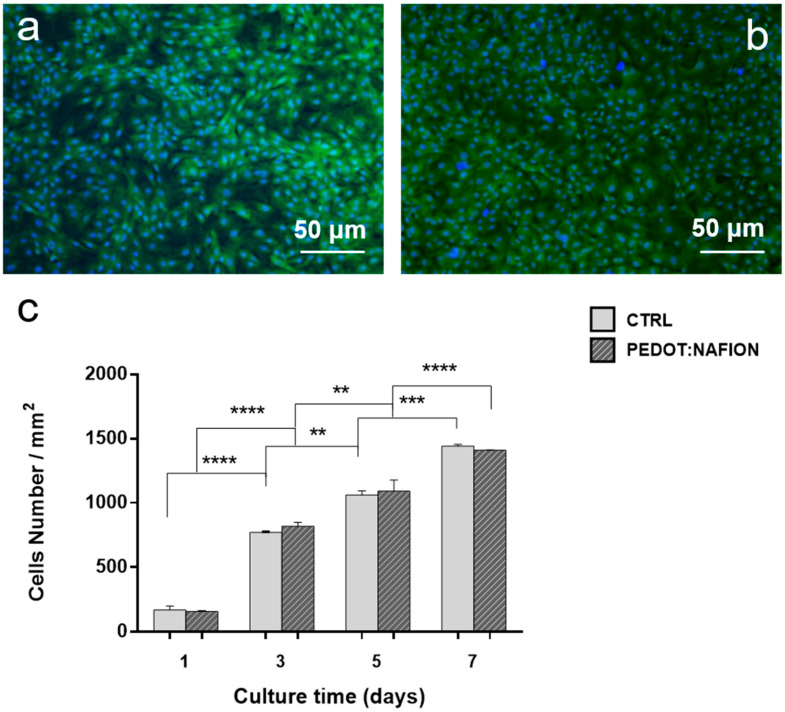
Cells proliferation on PEDOT:Nafion. Fluorescence images of primary rat fibroblasts stained with FDA (green) and Hoechst (blue) after 5 days from seeding on glass control (**a**) and PEDOT:Nafion coating (**b**); cell density (**c**) at different time points (** *p* < 0.01, *** *p* < 0.001, **** *p* < 0.0001).

The results of the proliferation assays are reported in Figure 4. Fluorescence images showed that fibroblasts grew rapidly on the PEDOT:Nafion coating, reaching complete confluence after 5 days (Figure 4a,b), similarly to what observed for fibroblasts seeded on the control group. Quantitative proliferation data obtained from cell nuclei counting indicated no statistical differences between the proliferation of cells on both experimental and control groups at the selected time points (Figure 4c).

### 3.3. Evaluation of Cell Viability on PEDOT:Nafion

**Figure 5:**
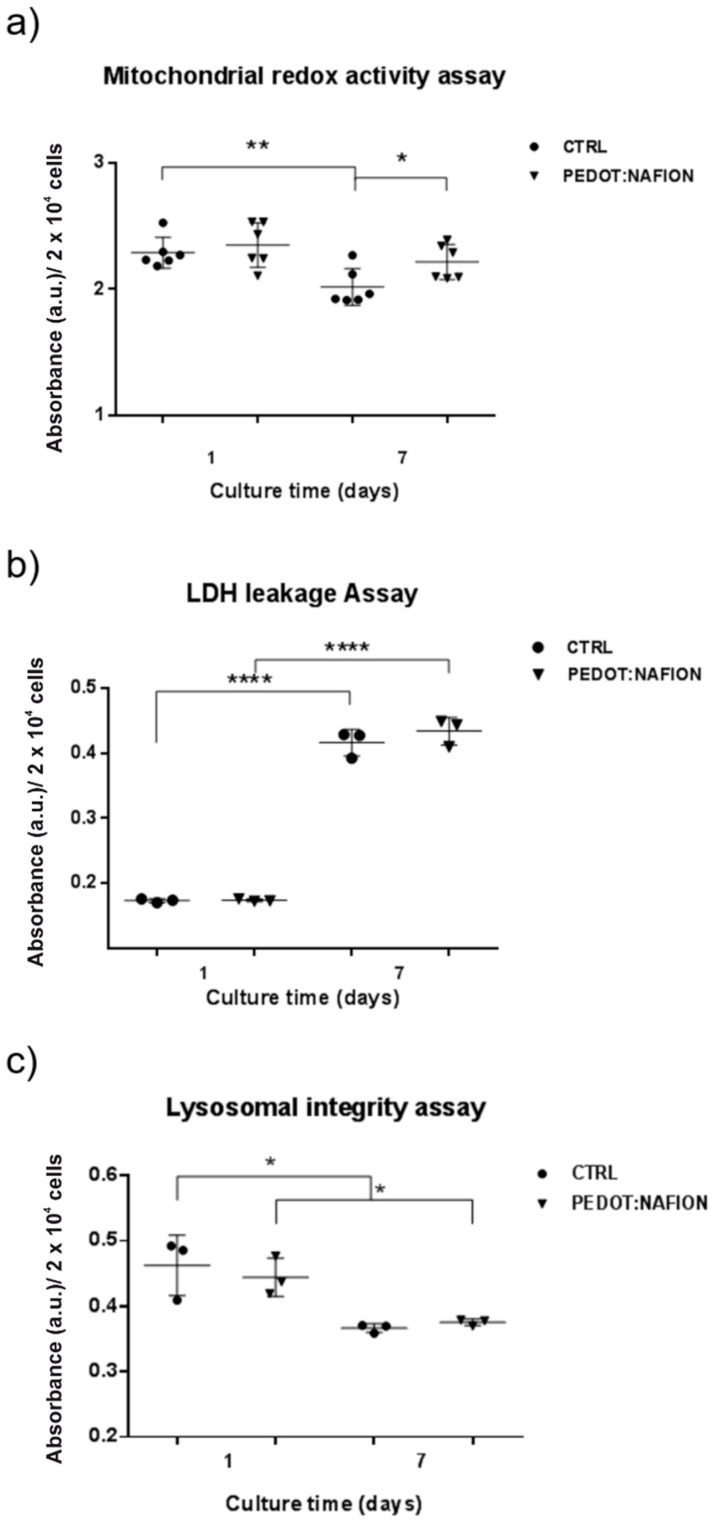
Evaluation of fibroblasts viability on PEDOT:Nafion coating. Cytotoxicity of the PEDOT:Nafion coating was evaluated by MTT (**a**), LDH (**b**) and neutral red assay (**c**) at 1 day and 7 days after seeding (* *p* < 0.05, ** *p* < 0.01, **** *p* < 0.0001).

MTT, lactate dehydrogenase (LDH) and neutral red assays were performed at 1 and 7 days in order to assess the mitochondrial redox activity, plasma membrane integrity and lysosomal activity, respectively, of fibroblasts seeded on PEDOT:Nafion coatings (Figure 5).

Mitochondrial redox activity was assessed by reduction of tetrazolium salt solution to formazan precipitate, which is impermeable to cell membranes and accumulates in viable cells, by mitochondrial succinate dehydrogenase in complex II [38,39]. As can be observed in Figure 5a, whereas cells viability was similar at day 1, cells cultured on PEDOT:Nafion showed higher viability than those proliferated on control at 7 days after seeding.

The LDH leakage test is based on the measurement of the release into the extracellular medium of the LDH enzyme (which converts lactate to pyruvate with subsequent reduction of NAD to NADH) after the disruption of the cell membrane [38,40]. The results of the assay indicated that LDH activity was similar for both the experimental and the control groups at day 1 and 7 (Figure 5b). In addition, LDH release increased for both groups from 1 to 7, likely due to initial degradation of the plasma membrane once the cells reached the confluence phase. Actually, cell density has been reported as a crucial factor in altering tests for cell viability, toxicity and apoptosis [41]. At confluence, probably reached at 7 days, fibroblasts had stopped dividing due to cell crowding, shape change and growth factors depletion. Considering that 7 days is quite a long period of incubation time, cells with a rapid proliferation rate such as rat fibroblasts should be seeded at the lowest density compatible with their growth so that confluence cannot be reached before the assay endpoint. In this regard, each assay showed different sensitivity, with LDH leakage being the most sensitive in detecting detrimental effects of cell confluence at 7 days compared to the neutral red and the MTT assay, as already observed elsewhere [15]. However, the similar results obtained for the PEDOT:Nafion coatings and the control substrate at each experimental time point support the conclusion that the fibroblasts are expressing normal functionality.

The neutral red cytotoxicity assay is a chemosensitivity test for cell viability based on the retention of a weakly cationic dye by the lysosomes of intact cells. Thus, the amount of released/retained dye is a direct marker of cell viability [17]. As can be observed in Figure 5c, no difference was found between the viability of fibroblasts grown on PEDOT:Nafion coatings and control substrate. Similar to what observed in the LDH assay, cell viability slightly decreased from 1 to 7 days, likely due to the achievement of the confluence stage. Taken together, the results of the MTT, LDH and neutral red assays indicate the PEDOT:Nafion were not cytotoxic under the investigated conditions.

## 4. Conclusions

In this study, we investigated for the first time the in vitro biocompatibility of novel PEDOT:Nafion conductive coatings fabricated starting from an aqueous dispersion of the polymer. PEDOT:Nafion has already demonstrated interesting electrochemical characteristics for field of organic bioelectronics, especially since it does not need any additive to improve adhesion or stability of the coating, which may adversely impact the biocompatibility of the material. The results reported in this work clearly indicate that PEDOT:Nafion coatings are not cytotoxic when tested with primary rat fibroblasts. In particular, thanks to their multiscale roughness, PEDOT:Nafion coatings are able to promote well cell adhesion and proliferation on their surface. These results support further investigation of PEDOT:Nafion dispersion as a viable alternative to the widely used PEDOT:PSS dispersion, especially in view of chronic in vivo applications.

## Data Availability

The data generated during and/or analyzed during the current study are available from the corresponding author on reasonable request.
